# Hepatic Radiotherapy in Addition to Anti-PD-1 for the Treatment of Metastatic Uveal Melanoma Patients

**DOI:** 10.3390/cancers15020493

**Published:** 2023-01-13

**Authors:** Ernesto Rossi, Francesco Cellini, Monica Maria Pagliara, Maria Grazia Sammarco, Romina Rose Pedone, Valentina Lancellotta, Luca Tagliaferri, Michela Quirino, Maria Antonietta Gambacorta, Maria Antonietta Blasi, Giampaolo Tortora, Giovanni Schinzari

**Affiliations:** 1 Medical Oncology, Fondazione Policlinico Universitario Agostino Gemelli IRCCS, 00168 Rome, Italy; 2 Radioterapia Oncologica ed Ematologia, Dipartimento di Diagnostica per Immagini, Fondazione Policlinico Universitario Agostino Gemelli IRCCS, 00168 Rome, Italy; 3 Radioterapia Oncologica ed Ematologia, Dipartimento Universitario Diagnostica per Immagini, Università Cattolica del Sacro Cuore, 00168 Rome, Italy; 4 Ophtalmology, Fondazione Policlinico Universitario Agostino Gemelli IRCCS, 00168 Rome, Italy; 5 Medical Oncology, Università Cattolica del Sacro Cuore, 00168 Rome, Italy

**Keywords:** uveal melanoma, liver metastases, immunotherapy, hepatic radiotherapy, anti-PD-1, immune checkpoint, liver directed therapies, pembrolizumab, tebentafusp

## Abstract

**Simple Summary:**

Uveal melanoma often metastasizes to the liver. Immune checkpoint inhibitors showed low efficacy in this disease. Liver directed therapies are widely employed despite limited results. The addition of hepatic radiotherapy to anti-PD-1 could enhance the efficacy of immune checkpoint inhibitor alone. In this study, efficacy and safety of radiotherapy on liver metastases combined with pembrolizumab have been retrospectively analyzed in previously untreated metastatic patients. This combination allowed encouraging results without increasing toxicity of anti-PD-1. Therefore, hepatic radiotherapy and anti-PD-1 can be considered a valid choice for untreated HLA A 02:01 negative patients as well as for second line systemic therapy after tebentafusp. Prospective trials should be conducted to confirm these observations.

**Abstract:**

Uveal melanoma is the most common ocular tumor with frequent metastatic spread to the liver. Immune checkpoint inhibitors have demonstrated poor results in this disease. The addition of hepatic radiotherapy to anti-PD-1 could enhance the sensitivity to immunotherapy. In this study, patients treated with pembrolizumab and who have undergone hepatic radiotherapy have been retrospectively evaluated. Twenty-two patients have been considered. Six patients (27.3%) achieved a partial response and 3 (13.6%) a stable disease. Disease control rate was 40.9%. Thirteen patients (59.1%) had progression as best response. The median PFS was 4.8 months and 6 months PFS rate 45.4%. The median OS was 21.2 months, while 1 year OS rate was 72.7%. Longer survival was observed in patients who achieved a partial response on irradiated metastases (HR 0.23, 95% CI 0.06–0.83) or progressed after 6 months (HR 0.12—95% CI 0.03–0.44). No radiotherapy-related or grade 3–4 adverse events were reported. This study demonstrates that the addition of hepatic radiotherapy to anti-PD-1 treatment can be a valid option for the treatment of metastatic uveal melanoma, particularly for HLA A 02:01 negative patients. Prospective studies should be conducted to confirm these data.

## 1. Introduction

Uveal melanoma is the most common ocular tumor. Metastases occur in up to half of patients, despite the radical treatment of primary melanoma. The most frequent site of metastases is the liver [1].

Both chemotherapy and immune checkpoint inhibitors (ICIs) showed limited results in advanced disease [1,2,3,4]. Other agents failed to demonstrate an improvement on clinical outcome [5,6,7,8]. A modest benefit has also been reported with the association of nivolumab and ipilimumab (PFS 3.0 months, OS 12.7 months) [9]. The poor results obtained with anti-PD-1 and anti-CTLA-4 agents in metastatic uveal melanoma can be explained by the features of the eye considered a “privileged immunological site”, the immune suppressive microenvironment of the liver, the low mutational burden, as well as further mechanisms of immune escape [10,11,12]. Moreover, liver metastases are associated with a lower efficacy of immunotherapy [13].

Tebentafusp, a bispecific gp100/anti-CD-3 fusion protein [14,15], is the only drug tested in a phase III study demonstrating a survival benefit for HLA A 02:01 positive metastatic uveal melanoma patients [16]).

Liver directed therapies (LDTs) are largely employed considering that hepatic disease is usually predominant in advanced uveal melanoma. Although selected patients may benefit from local treatment [17], none of LDTs has allowed robust results [1]. The combination of LDTs and ICIs could improve the clinical outcome in metastatic uveal melanoma patients, allowing a systemic disease control and reducing the risk of hepatic failure. Conventional radiotherapy (RT) and stereotactic body radiation therapy (SBRT) are included into the treatment algorithm of metastatic uveal melanoma [18]. Indeed, RT plays a relevant role for palliative treatment of metastatic cancer, such as in management of visceral, skeletal, and brain metastases [19]. Moreover, RT can reduce tumor growth outside the irradiated field. This effect, called “abscopal” [20], could be explained by radiation-induced cancer cell death, cytokines, damage-associated molecular patterns (DAMPs), and neoantigens generated by radiotherapy [21]. Therefore, RT can trigger anti-tumor immune surveillance, i.e., making tumor visible to the immune system [22,23]. An enhancement of anti-CTLA-4 and anti-PD-1 efficacy in melanoma patients has been observed with various RT fractionation schedules, mostly hypofractionated RT, which is usually employed for local control or palliative purpose [24,25]. In addition, low dose radiotherapy can modulate the tumor microenvironment of liver metastases, inducing recruitment and infiltration of the effector T cells [26].

In metastatic uveal melanoma, the addition of radiotherapy to anti-PD-1 agent could be a way to improve the clinical outcome, enhancing the benefit of the ICI and reducing the risk of hepatic failure.

In this study, we investigated efficacy and toxicity of radiotherapy in addition to anti-PD-1 treatment in previously untreated patients affected by metastatic uveal melanoma.

## 2. Materials and Methods

**Figure 1:**
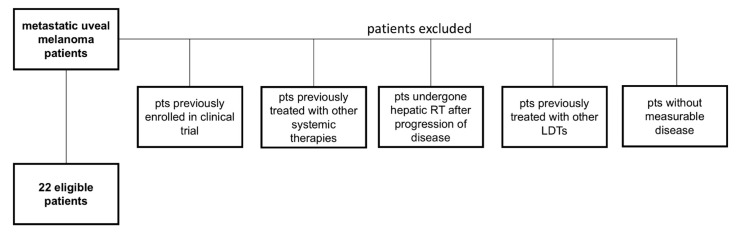
Patients’ selection process. The main characteristics excluding patients from the analysis are indicated. Pts: patients; RT: radiotherapy; LDTs: liver directed therapies.

In this retrospective analysis, the medical records of all metastatic uveal melanoma patients treated at Fondazione Policlinico Agostino Gemelli IRCCS were reviewed. Previously untreated subjects with histologically confirmed metastatic uveal melanoma were considered. In the analysis, we included patients treated with pembrolizumab as first line systemic therapy and undergone radiation therapy on symptomatic metastases or dominant hepatic metastatic sites, defined as metastases at high risk of local progression or life-threatening complications (i.e. biliary obstruction). Patients were included in the study if they also met the following criteria: age ≥ 18 years, Eastern Cooperative Oncology Group (ECOG) performance status of 0–2, at least 1 measurable metastasis according to Response Evaluation Criteria in Solid Tumors (RECIST) version 1.1. Figure 1 illustrates the patients’ selection process. Patients previously enrolled in clinical trials, already treated with systemic therapies or LDTs, undergone hepatic RT after progression of disease and without measurable disease were excluded.

All eligible patients received pembrolizumab at flat dose of 200 mg every 21 days and underwent radiation therapy during the period of treatment with the anti-PD-1 agent. RT on liver metastases was administered by hypofractionated schedule: 24 Gy total dose, 8 Gy per fraction -/fx-, along consecutive days. RT was performed through intensity modulated radiation therapy (IMRT). The hypofractionated palliative approach was applied to reduce risk of radio-induced toxicity. Three fractions of 8 Gy has been suggested to improve the efficacy of immune checkpoint inhibitors, particularly with administration of ipilimumab for metastatic melanoma [27,28]. Image guided radiation therapy (IGRT) was applied for all the patients through cone beam computed tomography scan. Respiratory gating was also applied in breath hold (BH) inspiration. Patients not compliant to BH received wider target margins and free-breathing (FB) respiratory gating. Margins for the clinical target volume to determine planning target volume accounted for 5 mm and 8 mm (isotropic) for the BH and FB, respectively.

In this retrospective study, the following clinical outcomes were analyzed: median progression-free survival (PFS), PFS rate at 6 months, overall survival (OS), OS rate at 1 years, response rate, toxicity.

PFS was calculated from the first day of pembrolizumab administration to progression or death for any cause. Survival was calculated from the first day of systemic therapy to death for any cause. PFS and OS were analyzed with the Kaplan–Meier method. The differences between patients’ subgroups were analyzed with the Log-rank test. Cox proportional-hazard model has been employed to calculate the hazard ratio for overall survival. Response was assessed according to RECIST version 1.1: complete response: disappearance of all target lesions; partial response (PR): more than 30% decrease of the sum of the longest diameter of all target lesions; progressive disease (PD): more than 20% increase of the sum of the longest diameter of target lesions (absolute increase ≥ 5 mm) or appearance of new lesions; stable disease: none of the above; disease control rate: percentage of patients with CR + PR + SD. Toxicity was evaluated with Common Terminology Criteria for Adverse Events (CTCAE) version 6.0.

## 3. Results

Twenty-two patients who received both hepatic RT and anti-PD-1 from October 2017 to December 2021 were identified. Patients’ characteristics are described in Table 1. Median age was 65.4 years (range 51–85). All the patients had liver metastases, and 10 of them (45.4%) also had extrahepatic disease. Enucleation has been previously performed in 16 patients. Radiation therapy was carried out during the interval between pembrolizumab administrations. In Table 2, irradiated fields are described.

Six patients (27.3%) achieved a partial response and 3 (13.6%) a stable disease. Disease control rate was 40.9%. Thirteen patients (59.1%) had progression as best response. Considering the objective response of irradiated metastases, partial response, stable disease and progression was observed in 8 (36.4%), 6 (27.2%) and 8 patients (36.4%), respectively.

**Figure 2:**
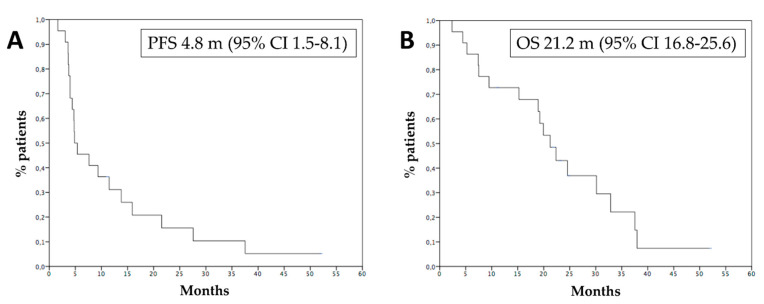
Progression-free survival (**A**) and overall survival (**B**) of the patients treated with hepatic radiotherapy and anti-PD-1. m: months.

**Figure 3:**
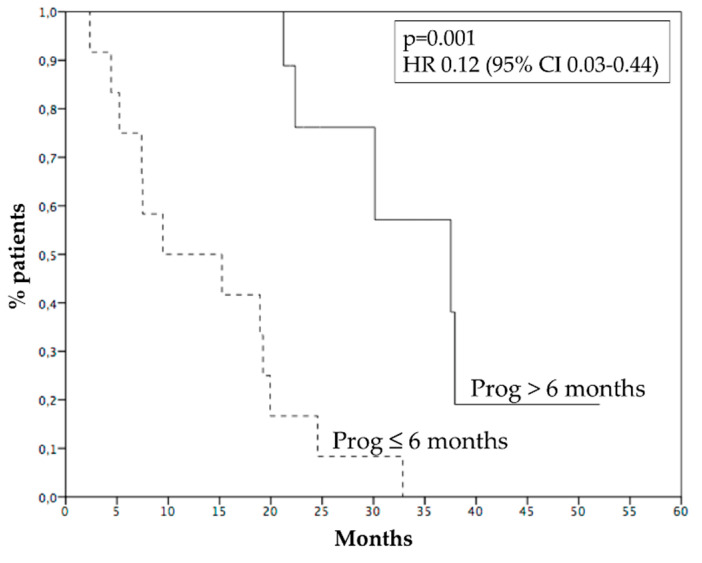
Survival of patients progressed after 6 months (37.5 months, solid line) and within 6 months (9.4 months, dashed line). Prog: progression.

**Figure 4:**
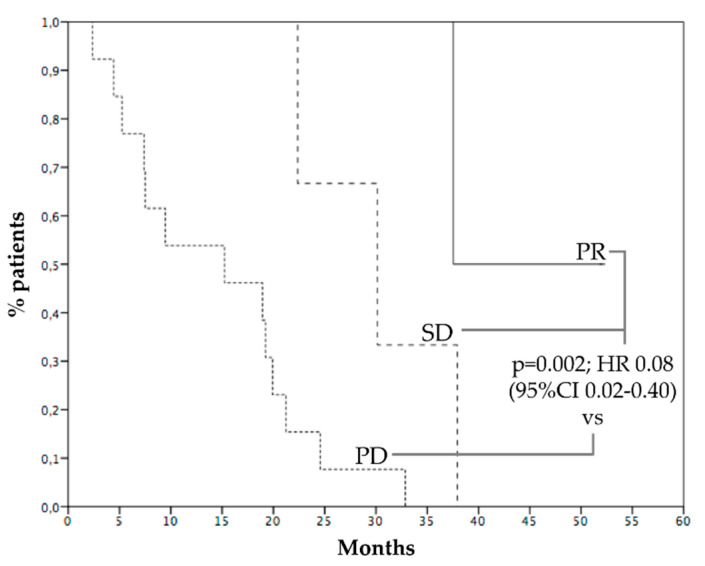
Survival according to response rate obtained with hepatic radiotherapy and anti-PD-1. PR: partial response (37.5 months, solid line); SD: stable disease (30.1 months, dashed line); PD: progressive disease (15.2 months, dotted line).

**Figure 5:**
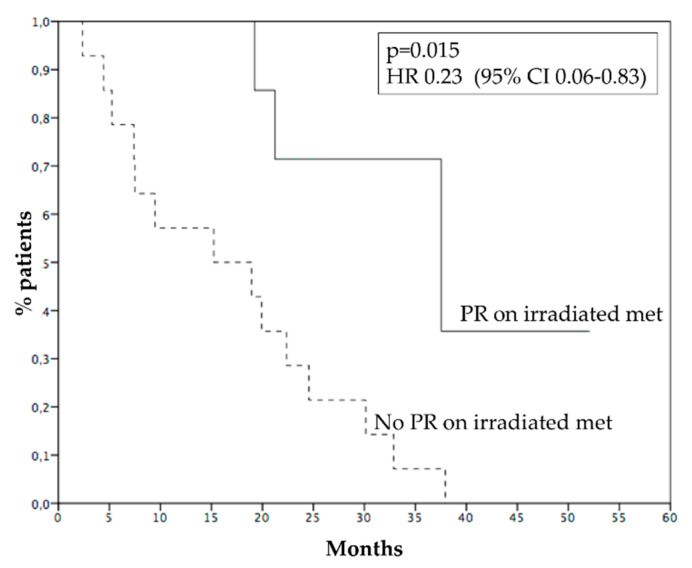
Survival of patients achieving a partial response of irradiate metastases (37.5 months, solid line) or not (15.2 months, dashed line).

**Figure 6:**
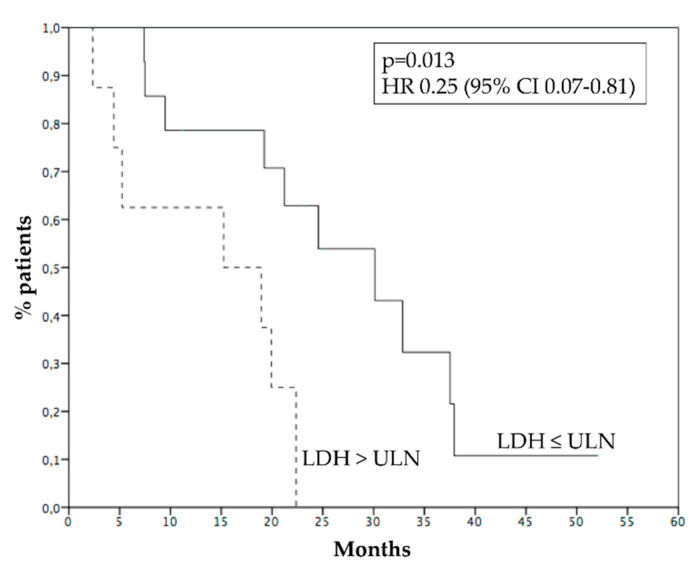
Survival according to basal LDH: normal (30.1 months, solid line) or higher than ULN (15.2 months, dashed line). ULN: upper limit of normal.

Median PFS was 4.8 months (Figure 2A), 6 months PFS rate 45.4%. Median OS was 21.2 months (Figure 2B), while 1 year OS rate was 72.7%. Survival of patients who progressed within 6 months was 9.4 months, while survival of patients who progressed after 6 months was 37.5 months (*p* = 0.001, HR 0.12—95% CI 0.03–0.44, Figure 3). Figure 4 reports survival according to response rate: OS was 37.5 months for patients with partial response, 30.1 months for patients with stable disease, and 15.2 months for patients with progression as the best response. Patients obtaining a partial response of irradiated metastases had a better survival (37.5 months) than patients without objective response of irradiated metastases (15.2 months) (*p* = 0.015, HR 0.23, 95% CI 0.06–0.83—Figure 5). OS of patients with baseline lactate dehydrogenase (LDH) higher than upper limit of normal (ULN) was 15.2 months, OS of patients with normal LDH was 30.1 months (*p* = 0.013, HR 0.25—95% CI 0.07–0.81, Figure 6).

A multivariate analysis was performed to test the influence on survival of LDH and objective response of irradiated metastases (Table 3). A reduction in risk of death was shown for both the variables, with upper confidence limit slightly higher than 1. Univariate analysis was also performed to test the combined effect of normal LDH and objective response to RT. The hazard ratio for survival considering together LDH ≤ ULN and objective response to RT was 0.11 (95% CI 0.02–0.48) in comparison with LDH > ULN and no response to RT.

No radiotherapy-related or grade 3–4 adverse events were reported. Hypothyroidism was observed in 3 patients, 2 of them with grade 1 and 1 with grade 2. Grade 1 rash was reported in 1 patient, grade 1 diarrhea in another patient.

## 4. Discussion

Uveal melanoma is a rare tumor with few treatment options for metastatic disease. Data from prospective studies are limited and the results obtained from most of the available treatments are not encouraging. Even the combination of nivolumab and ipilimumab demonstrated modest activity, with median OS and PFS of 12.7 months and 3.0 months, respectively. [9]. This combination therapy was also tested in the study conducted by Pelster, in which OS was 19.1 months and PFS 5.5 months [29]. Similar results were observed with treatment strategies different from ICIs (i.e., chemotherapy or LDTs). Recently, tebentafusp allowed a better survival versus single ICI (pembrolizumab or ipilimumab) or chemotherapy, with 1 year survival rate of 73% versus 59% of the control arm. Tebentafusp has also improved the progression free survival (31% vs. 19% at 6 months). Unfortunately, these advantages are limited to HLA A 02:01 positive patients [30], who represent up to 45% of metastatic uveal melanoma patients. No standard therapy has yet been established for HLA A 02:01 negative patients.

Uveal melanoma is characterized by hepatic spread. Liver failure is often the cause of death. Various LDTs have been employed, aiming to gain tumor shrinkage and delay loss of hepatic function. Among LDTs, experiences with several approaches have been reported, such as surgery, isolated hepatic perfusion, hepatic artery infusion, transarterial chemoembolization, selective internal radiotherapy, and immunoembolization [17]. Nevertheless, none of LDTs demonstrated impressive benefit for metastatic uveal melanoma patients. ICIs are less effective when liver metastases are present. Several uveal melanoma features can explain the poor response to ICIs.

In clinical practice, the need of more effective treatments has led to combine a systemic therapy with a LDT, considering the potential ability to enhance clinical benefit of ICIs. This strategy could improve overall survival, as demonstrated by retrospective analyses [31,32]. In particular, the combination of LDTs and ICIs leads to a longer survival compared to LDTs/systemic therapies only [31]. Promising survival rates with sequential dual ICIs and selective internal radiotherapy have been observed, with a 44.4% of grade 3–4 adverse events [33]. Radiotherapy represents another treatment directed to the liver which can allow a local control with an acceptable tolerability [24,25].

In the present study, metastatic uveal melanoma patients undergone liver radiotherapy while receiving pembrolizumab as first line therapy were considered. Median PFS was 4.8 months with a 6 months PFS rate of 45.4%. Median OS was 21.2 months, with a 1 year OS rate of 73%. These results are similar to the data reported with tebentafusp in HLA A 02:01 population. The addition of hepatic RT to anti-PD-1 seems to improve the clinical outcome of ICI only. Indeed, it has been reported a PFS of 3.8 months with pembrolizumab [3]. In a retrospective analysis with nivolumab, PFS and an OS were 10 and 60 weeks, respectively. [34]. The prospective trial testing tebentafusp showed a PFS of 2.9 months and an OS of 16 months in the control arm (including pembrolizumab or nivolumab or dacarbazine) [16].

This study has also demonstrated a correlation between tumor control and survival. Indeed, OS was longer in patients with partial response or stable disease compared to patients with progression as best response. Tumor reduction in irradiated sites was also associated with longer survival. Similarly, the phase 3 study of tebentafusp showed a longer survival for patients gaining tumor regression. It has been also described a longer survival in case of objective responses to ICIs [35]

In the present analysis, patients were treated with non-ablative RT, receiving doses able to achieve palliation or mild local control. It has been previously reported the ability of low dose RT to module the liver environment, favoring a more efficacy of immunotherapy [26]. An improvement of overall survival has been observed in cutaneous melanoma patients treated with a local peripheral treatment, including RT, and immunotherapy [25]. A portion of these results have been explained with an abscopal effect [25]. The association of pembrolizumab and hepatic RT was feasible and safe, without additional toxicity on what expected from anti-PD-1 alone. All the patients underwent RT at least on one liver metastasis. Some patients received RT on more than one hepatic lesions, without complications. Therefore, liver directed radiotherapy can be also considered an option to treat oligoprogressive hepatic disease in association to anti-PD-1, administered beyond progression [36].

Normal LDH has been associated with longer survival in metastatic uveal melanoma [16,37]. Our analysis has confirmed the prognostic role of basal LDH for metastatic uveal melanoma, reinforcing the need to establish a more effective treatment for patients with poor prognosis.

The present study is limited by the small number of patients, the absence of a control cohort, and the retrospective design. Nevertheless, this study demonstrates that the combination of hepatic RT and anti-PD-1 could be a valid option for the treatment of metastatic uveal melanoma, particularly for patients without HLA A 02:01. In patients with HLA A 02:01, radiotherapy plus anti-PD-1 can also allow a further benefit after tebentafusp. Hepatic RT and anti-PD-1 may be considered mainly when the metastases are limited to the liver or in case of predominant hepatic disease. The identification of predictive factors for immunotherapy, such as circulating factors involved in immune response [38], can select patients who could benefit more from this combined treatment.

## 5. Conclusions

This study suggests that hepatic radiotherapy combined to anti-PD-1 could represent a valid and safe option to improve the results obtained with ICI alone. This strategy can be considered as first line systemic therapy for HLA A 02:01 negative patients or as second line therapy after tebentafusp for HLA A 02:01 positive uveal melanoma patients. Prospective studies should be conducted to confirm these data.

## Figures and Tables

**Table 1:** Patients’ characteristics.

	N. (%)
M/F	7/15
Median age (range)	65.4 y (51–85)
ECOG 0–1–2	10 (45.4)–8 (36.4)–4 (18.2)
Liver metastases	22 (100)
Extrahepatic metastases	10 (45.4)
Enucleation for primary tumor	16 (72.7)
LDH > ULN	8 (36.4)

y: years; ULN: upper limit of normal.

**Table 2:** Metastatic sites treated with radiotherapy.

Patients	Hepatic Segments	Volume (cm^3^)
#1	S 8	6.51
#2	S 7–8	12.64
#3	S 4–5–6	22.2
#4	S 3–4	4.41
#5	S 4	3.43
#6	S 5–6; S 8	4.43; 5.2
#7	S 5; S 6	5.04; 5.71
#8	S 2; S 4	3.91; 4.49
#9	S 1	17.56
#10	S 6–7	3.52
#11	S 2; S 8	19.62; 14.7
#12	S 4	309.6
#13	S 4–5	27.3
#14	S 5	8.36
#15	S 8	204.0
#16	S 5–6	37.38
#17	S 4	10.75
#18	S 4; S 8	8.91; 14.1
#19	S 8	37.4
#20	S 6	2.16
#21	S 8	3.64
#22	S 4	5.82

**Table 3:** Cox Regression of survival according to LDH and response to RT.

	Hazard Ratio (HR)	Confidence Interval (CI) 95%
**Multivariate analysis**		
**LDH**		
>ULN	1	-
≤ULN	0.34	0.10–1.13
**Response to RT**		
No	1	-
Yes	0.29	0.08–1.05
**Univariate analysis**		
LDH > ULN AND No Response	1	-
LDH ≤ ULN OR Response	0.25	0.07–0.87
LDH ≤ ULN AND Response	0.11	0.02–0.48

## Data Availability

The data presented in this study are available on reasonable request from the corresponding author.

## Abbreviations

ICIs	Immune Checkpoint Inhibitors
PFS	Progression-Free Survival
OS	Overall Survival
LTDs	Liver Directed Therapies
RT	Radiotherapy
SBRT	Stereotactic Body Radiation Therapy
DAMPs	Damage-Associated Molecular Patterns
ECOG	Eastern Cooperative Oncology Group
RECIST	Response Evaluation Criteria in Solid Tumors
IMRT	Intensity Modulated Radiation Therapy
CTCAE	Common Terminology Criteria for Adverse Events
LDH	Lactate Dehydrogenase
ULN	Upper Limit of Normal
PR	Partial Response
SD	Stable Disease
PD	Progressive Disease
IGRT	Image guided radiation therapy
BH	Breath Hold
FB	Free-Breathing
